# Metastatic Cutaneous Squamous Cell Carcinoma to the Axilla: A Review of Patient Outcomes and Implications for Future Practice

**DOI:** 10.4021/wjon503w

**Published:** 2012-10-28

**Authors:** Nadine Beydoun, Peter H Graham, Lois Browne

**Affiliations:** a Department of Radiation Oncology, St George Hospital, Kogarah, New South Wales, Australia; b University of New South Wales, Sydney, New South Wales, Australia

**Keywords:** Axilla, Metastatic, Radiotherapy, Skin cancer, Squamous cell carcinoma

## Abstract

**Background:**

Nodal metastasis from cutaneous SCC carries a high morbidity and mortality. Limited direct evidence is available as to the impact of radiotherapy on the outcome of patients with metastatic axillary SCC. The purpose of this study was to report on the outcomes of patients with metastatic cutaneous SCC to the axilla treated with radiotherapy.

**Methods:**

A retrospective review of patients treated with radiotherapy between 1993 and 2010 for metastatic cutaneous SCC to the axilla was undertaken at St George Hospital, Sydney.

**Results:**

Radiotherapy was administered to 36 patients, 30 with curative intent (4 definitive, 26 adjuvant) and 6 with palliative intent, 27/36 (75%) were male, 22/36 (61%) had a previous diagnosis of cutaneous SCC, and 1/36 (3%) was immunosuppressed. Mean age was 74.6 years. Mean radiotherapy dose (BEDGy10) was 61Gy_10_ (range 39-85 Gy_10_), 20/36 (56%) patients experienced recurrence, including 16 local failures and 4 isolated distant failures. Median survival for the curative and palliative groups was 3 years and 1 month, respectively. Relapse free survival (n = 36) at 2 and 5 years was 46% and 35%, respectively (curative 52% and 39%). Only 1 failure achieved complete salvage.

**Conclusion:**

Despite current best practice (surgery and radiotherapy), the predominant pattern of failure in these patients with metastatic axillary cSCC was locoregional. The difficulty in successfully salvaging patients after locoregional nodal relapse suggests a need for treatment intensification.

## Introduction

Non-melanoma skin cancer (NMSC) is the commonest malignancy worldwide. The highest incidence of NMSC is in Australia, with two in every three Australians developing a skin cancer in their lifetime. The majority of NMSCs arise on sun-exposed areas, including the head and neck region (70-80%), most of which are basal cell carcinomas (BCCs). Cutaneous squamous cell carcinoma (cSCC) comprises 20% of all NMSC [1]. Most are highly curable with surgery and/or radiotherapy, but a small proportion will metastasise (< 5%) [2].

Metastatic cSCC carries a poor prognosis, and patients with uncontrolled locoregional recurrence will ultimately succumb to this disease. A number of studies have documented improved locoregional control (LRC) rates with surgery and radiotherapy in the setting of metastatic cSCC to parotid or cervical LNs, and this has become the standard of care [3]. Limited evidence however is available that describes outcomes for metastatic cSCC to the axilla including radiotherapy.

The purpose of this study was to report on the outcomes of patients with metastatic cSCC to the axilla treated with radiotherapy at one institution, and to discuss the implications for future management of this disease.

## Materials and Methods

A retrospective review of patients with metastatic cSCC to the axilla treated with radiotherapy between 1993 and 2010 at St George Hospital was undertaken. Relevant details regarding patient demographics, treatment, and outcome were obtained using a computer database and cancer care centre files. Patients who died prior to undertaking radiotherapy were excluded. Toxicity scores used Common Terminology Criteria for Adverse Events (CTCAE) version 3 [4]. Factors associated with skin toxicity and radiotherapy treatment interruption were analysed using Chi-Square test, Fisher’s test or logistic regression. Local recurrence was defined as disease recurrence within the axilla, and was measured from the date of first intervention. All other recurrences were classified as distant. Local control, relapse free survival (RFS), and overall survival (OS) were estimated using the method of Kaplan-Meier, and Hazard Ratios (HR) obtained using Cox Proportional Hazards modelling. Patients were censored at the time of last follow-up or death. Elapsed times were calculated from the date of first intervention (date of surgery or date of radiotherapy commencement for those treated with radiation only). Variables considered a priori to be potential predictors of outcome were use of bolus, single modality treatment, size (greater or less than 3 cm), number of nodes (single versus multiple), extracapsular extension, radiotherapy dose, early cessation of radiotherapy and time from surgery to start of radiotherapy. Data were analysed using STATA-SE version 10.

## Results

### Patient and tumor characteristics

**Figure 1:**
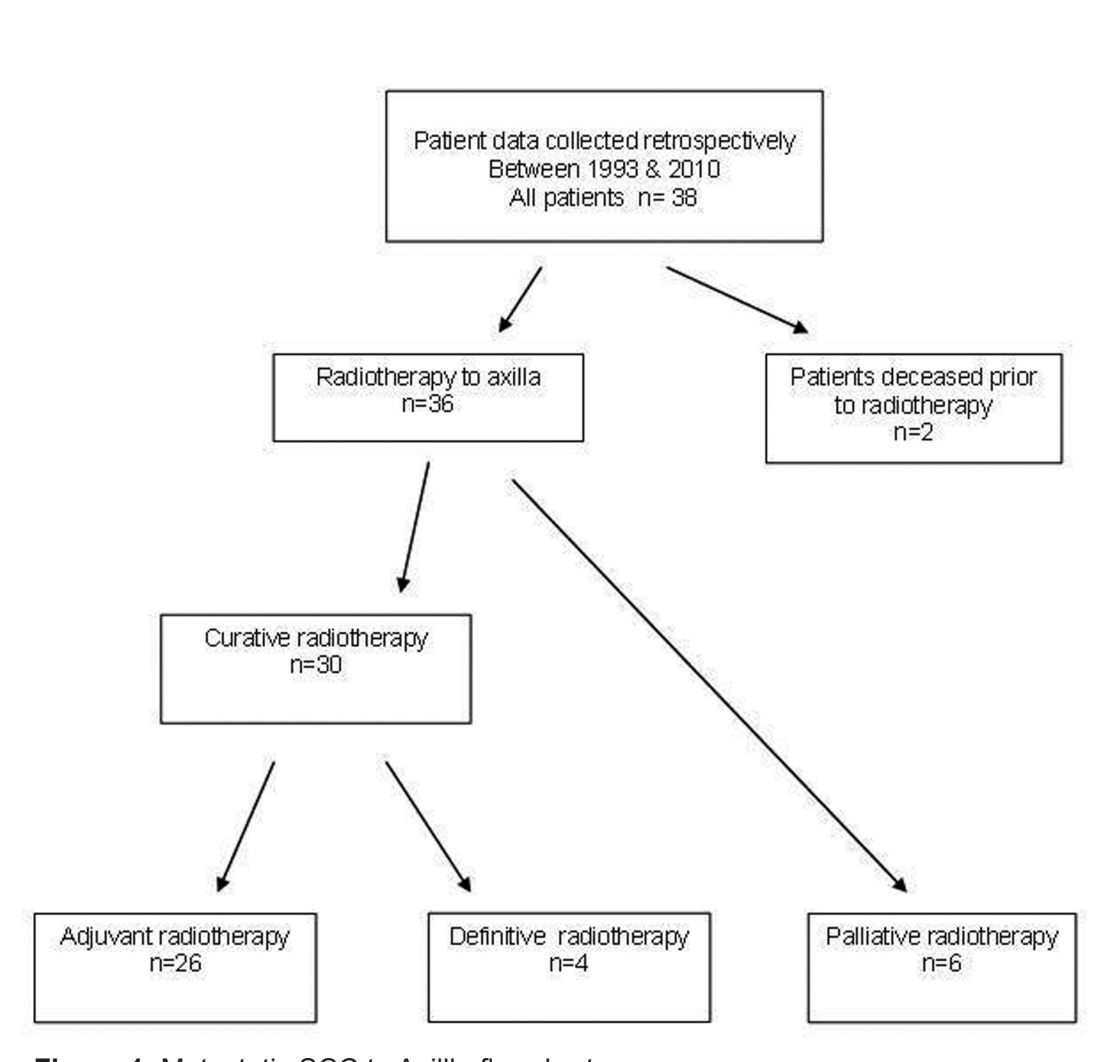
Metastatic SCC to Axillla flowchart.

A total of 36 patients were treated with radiotherapy for metastatic cSCC to the axilla, 30 with curative (26 surgery and radiotherapy, 4 definitive radiotherapy) and 6 with palliative intent (Fig. 1). Patient demographics, tumor characteristics and surgery details are presented in Table 1. Median follow-up time was 3.2 years for the curative group (range 0.3 - 12.4) and 0.25 years (range 0.11 - 0.46) for the palliative group. The majority of patients (61%) had a previous diagnosis of non-melanoma skin cancer. All patients were ECOG 0-2. One patient was immunosuppressed due to renal transplantation. Staging routinely included CT scanning but not PET scans as these are not re-imbursed for this indication.

**Table 1:** Patient and Tumor Demographics

	Adjuvant (n = 26)	Definitive (n = 4)	Palliative (n = 6)	Total (n = 36)
Mean age (range)	75 (36 - 92)	74 (63 - 86)	73 (58 - 86)	75 (36 - 92)
Gender (%)				
Male	21 (81)	3 (75)	3 (50)	27 (75)
Female	5 (19)	1 (25)	3 (50)	9 (25)
Smoker (%)				
Yes	1 (4)	0 (0)	0 (0)	1 (3)
Ex	7 (27)	2 (50)	4 (67)	13 (36)
No	15 (58)	2 (50)	2 (33)	19 (53)
Unknown	3 (11)	0 (0)	0 (0)	3 (8)
ECOG Performance status (%)				
0	13 (50)	1 (25)	0 (0)	14 (39)
1	7 (27)	2 (50)	4 (67)	13 (36)
2	6 (23)	1 (25)	2 (33)	9 (25)
Previous non-melanoma skin cancer (%)				
Yes	15 (58)	3 (75)	4 (67)	22 (61)
No	11 (42)	1 (25)	2 (33)	14 (39)
Location of Index Lesion (%)				
Head and neck	1 (4)	0 (0)	4 (66)	5 (14)
Trunk	12 (46)	1 (25)	1 (17)	14 (39)
Upper Limb	8 (31)	2 (50)	0 (0)	10 (28)
Unknown	5 (19)	1 (25)	1 (17)	7 (19)
Size of axilla mass (%)				
< 3 cm	9 (35)	1 (25)	0 (0)	10 (28)
3 - 6 cm	11 (42)	1 (25)	4 (67)	16 (44)
> 6 cm	6 (23)	2 (50)	2 (33)	10 (28)
Surgery (%)				
Axillary Dissection	21 (81)	0 (0)	1 (17)	22 (61)
Lumpectomy	5 (19)	0 (0)	0 (0)	5 (14)
None	0 (0)	4 (100)	5 (83)	9 ( 25 )
Histological Differentiation (%)				
Moderate	11 (42)	0 (0)	2 (33)	13 (36)
Poor	10 (39)	1 (25)	2 (33)	13 (36)
Unknown	5 (19)	3 (75)	2 (34)	10 (28)
Extracapsular extension (%)				
Yes	15 (58)	1 (25)	2 (33)	18 (50)
No	6 (23)	1 (25)	0 (0)	7 (19)
Unknown	5 (19)	2 (50)	4 (67)	11 (31)
Invasion of adjacent structures (%)				
Yes	3 (11)	2 (50)	3 (50)	8 (22)
No	22 (85)	2 (50)	2 (33)	26 (72)
Unknown	1 (4)	0 (0)	1 (17)	2 (6)

### Treatment of axillary nodal metastases

The majority of patients had surgery prior to radiotherapy (27/36; 75%), 22 undergoing axillary dissection and 5 lumpectomy only. Mean time from diagnosis to surgery in palliative and curative patients was 52 versus 12 days. The time from diagnosis to radiotherapy for the adjuvant, definitive and palliative groups, was 82 (range 18 - 183), 95 (range 42 - 190) and 63 days (range 11 - 206), respectively. Mean time from surgery to adjuvant radiotherapy was 71 days (range 29 - 183). Radiotherapy was delivered using an anterior-posterior field technique in 35/36 patients (97%). Only one patient was treated using a multifield conformal technique. Variation in volume, dose, fractionation and bolus use is detailed in Table 2. When applied, bolus was placed over surgical scars or gross disease involving skin. Mean radiation dose (BED (Biological Equivalent Dose) was 61Gy_10_ (range 47-85 Gy_10_) in the adjuvant, 70Gy_10_ (range 63-84Gy_10_) in the definitive, and 55Gy_10_ (range 39 - 72Gy_10_) in the palliative group (Table 2).

**Table 2:** Radiotherapy Details

	Adjuvant (n = 26)	Definitive (n = 4)	Palliative (n = 6)	Total (n = 36)
Axilla Dose (Gy) mean (range)	51 (38 - 70)	56 (50 - 70)	41 (30 - 50)	50 (30 - 70)
Fractions mean (range)	24 (15 - 33)	23 (18 - 35)	14 (5 - 20)	22 (5 - 35)
Bed Gy10 mean (range)	61 (47 - 85)	70 (63 - 84)	55 (39 - 72)	61 (39 - 85)
Treated sites n (%)				
Axilla	6 (23)	2 (50)	5 (83)	13 (36)
Axilla + SCF	15 (57)	2 (50)	1 (17)	18 (50)
Axilla + SCF + Primary	5 (20)	0 (0)	0 (0)	5 (14)
Bolus n (%)	12 (46)	1 (25)	1 (17)	14 (39)
SCF BED Gy_10_ if included mean (range)	58 (47 - 67)	55 (47 - 63)	29 (na)	57 (29 - 67)
SCF BED Gy_3_ if included mean (range)	82 (69 - 95)	82 (72 - 92)	59 (na)	81 (59 - 95)

### Skin toxicity

Grades 1, 2 and 3 skin toxicities at radiation completion (n = 36) were recorded in 11 (31%), 22 (61%), and 3 (8%) patients, respectively. Due to the limited numbers, grade 2 and 3 skin toxicities were combined for analysis. All 14 patients with bolus (12 adjuvant, 1 definitive, 1 palliative) experienced Grade > 1 skin toxicity. Grade > 1 skin toxicity was seen in 50% of patients with no bolus (11/22 total; 6/14 adjuvant, 1/3 definitive, 4/5 palliative). The use of bolus resulted in significantly worse skin toxicity at treatment completion (P = 0.002).

Of patients who had radiotherapy to the axilla and SCF (extended field), 18/23 (78%) experienced Grade > 1 skin toxicity (16/20 adjuvant, 1/2 definitive, 1/1 palliative). Of those with no SCF radiotherapy, 7/13 (54%) had grade > 1 skin toxicity (2/6 adjuvant, 1/2 definitive, 4/5 palliative). There was a trend towards greater skin toxicity with treatment of the axilla and SCF compared with axilla only, but this did not reach statistical significance (P = 0.13).

Mean BED Gy_10_ was 62 in patients with grade 2/3 skin toxicity, and 60 in those with grade 1 toxicity. These differences were not statistically significant.

Radiotherapy was ceased early in two patients. This was due to grade 3 skin toxicity in both cases. Both patients were treated with extended field adjuvant radiotherapy including scar bolus. Only one other patient had grade 3 skin toxicity, and this was in the setting of a hypofractionated radiotherapy course to the axilla (40Gy in 5 weekly fractions) and SCF (16Gy in 2 weekly fractions), one fraction per week. Bolus was not used in this instance, and the patient completed the full treatment course.

### Lymphoedema

Twenty eight percent (10/36) patients developed lymphoedema (6 adjuvant, 1 definitive, 3 palliative). Three patients had lymphoedema prior to radiotherapy: 2 presented with lymphedema at diagnosis and 1 post-operatively. Seven patients developed lymphoedema simultaneously with local recurrence. None of the remaining patients without recurrence developed lymphoedema.

### Plexopathy

Twenty five percent (9/36) patients developed brachial plexopathy symptoms (6 adjuvant, 3 definitive). Plexopathy co-incided with local recurrence in 8 of these. Of patients who did not have a recurrence recorded at the time of last follow-up, only 1 developed plexopathy symptoms. The dose received in this instance was 55Gy in 25 fractions to the axilla and SCF (BED Gy_3_ 116).

### Local recurrence

Only one patient had recurrence in the SCF, occurring simultaneously with recurrence in the axilla. Treatment in this case was 40Gy in 15 fractions to the axilla only (palliative). No patient had isolated SCF recurrence, and no patient in the curative group recurred in the SCF, irrespective of whether this was included in the radiation field.

In total 20/36 (56%) patients experienced a recurrence, 16 of which were local (9 adjuvant, 3 definitive, 4 palliative). Median time to local recurrence was 5 years overall: 6.1 years, 0.6 years , and 0.3 years in the adjuvant, definitive, and palliative groups, respectively. Local recurrence rates in the adjuvant radiotherapy group at 2 and 5 years were 31% (95%CI 16-55%) and 43% (95%CI 22-72%), respectively. In the 4 definitive radiotherapy patients, local recurrence rates at 2 and 5 years were 50% (95%CI 16-94%) and 75% (95%CI 33-99%), respectively.

Univariate time to event analysis was undertaken for the curative patients only (n = 30), as the number of events was small. No variables considered a priori to be potential predictors of time to local recurrence were found to be statistically significant.

### Distant recurrence

There were 7 patients who experienced distant recurrence, and all of these were in the adjuvant radiotherapy group. They included 1 patient who had a local recurrence prior to distant metastasis, and a further 2 who had simultaneous local and distant relapse. The remaining 4 patients had isolated distant recurrences. The median time to distant recurrence in the adjuvant group was 6.1years. The majority of distant recurrences were in the lung. Other sites included the contralateral axilla, groin and pelvis.

### Treatment of recurrence

Recurrences were treated with combinations of chemotherapy (platinum or 5-FU based), surgery, and radiotherapy. Surgery consisted of contralateral axilla dissection, pelvic and inguinal lymphadenectomy, and limb amputation. Of patients who experienced a recurrence, only 2 were long term survivors. The first had relapse in the axilla and lung that was treated with chemotherapy and was alive with stable disease 2 years later. The other had isolated relapse at the primary site and was treated with upper limb amputation. This patient was alive with no evidence of disease 8 years later.

### Survival

Table 3 shows 1, 2 and 5 year survival for each of the three treatment groups. Of the patients treated with curative intent (definitive + adjuvant), 11/30 died of metastatic cSCC, while 3/6 patients died of metastatic cSCC in the palliative group (total 14/36 patients).

**Table 3:** Relapse Free Survival and Overall Survival

		Adjuvant (n = 26)	Definitive (n = 4)	Palliative (n = 6)	Total (n = 36)
% Relapse Free Survival (95% CI)	1 yr	71 (49 - 85)	50 (6 - 84)	0	60 (41 - 75)
	2 yr	52 (29 - 70)	50 (6 - 84)		46 (27 - 62)
	5 yr	41 (18 - 64)	25 (0 - 67)		35 (16 - 53)
% Overall Survival (95% CI)	1 yr	75 (53 - 88)	50 (6 - 84)	0	64 (45 - 78)
	2 yr	58 (36 - 75)	50 (6 - 84)		50 (32 - 66)
	5 yr	32 (14 - 52)	25 (0 - 67)		27 (13 - 44)

**Figure 2:**
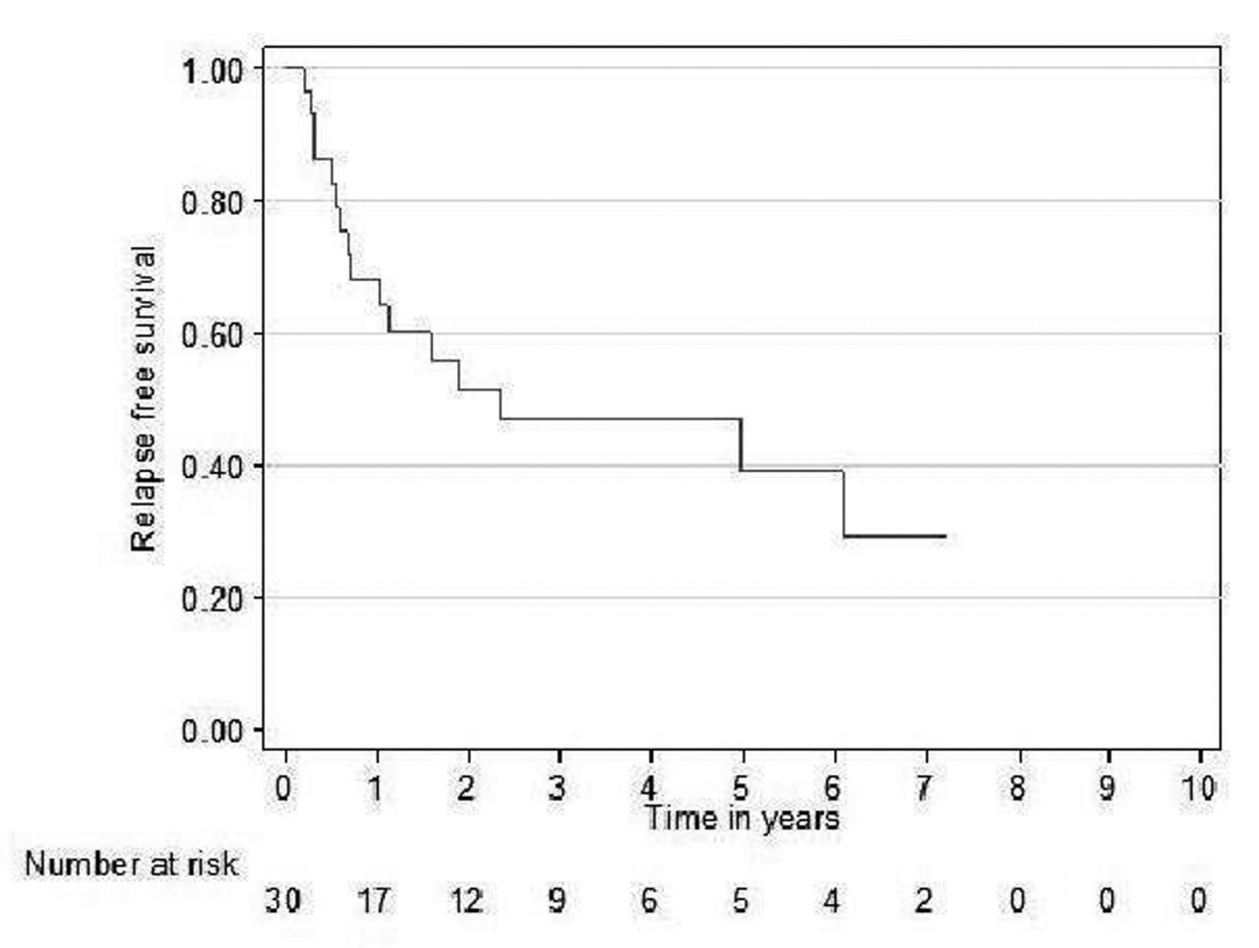
Relapse free survival curve for curative patients.

**Figure 3:**
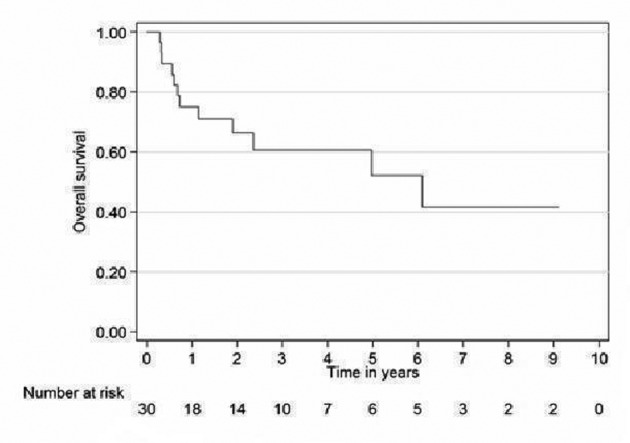
Overall survival curve for curative patients.

Relapse free survival rates for the curative patients at 2 and 5 years (adjuvant + definitive) were 52% (95% CI 31 - 69) and 39% (95% CI 19 - 59), respectively (Fig. 2). Overall survival rates for the curative patients at 2 and 5 years were 57% (95% CI 36 - 73) and 31% (95% CI 14 - 49, respectively (Fig. 3). No variables considered a priori to be potential predictors were found to be statistically significant.

## Discussion

In keeping with other series, the majority of patients in our study were elderly, male, and had a previous diagnosis of non-melanoma skin cancer [1, 5].

Our study was not designed to identify predictors of nodal presentation, but outcome following treatment for axillary nodal presentation. It has been demonstrated previously that immunosuppression is associated with poorer outcomes in patients with metastatic cSCC [6]. We were unable to draw a comparison in outcomes between immunocompromised and immunocompetent patients, however our single immunocompromised patient had isolated distant failure and died of metastatic cSCC.

The optimal radiotherapy dose and technique for treatment of metastatic cSCC to the axilla has not been evaluated in a prospective trial. Current National Comprehensive Cancer Network (NCCN) Guidelines recommend 60Gy in 30 fractions (BED Gy_10_ 72) following dissection with extracapsular extension and 54Gy in 27 fractions (BED Gy_10_ 65) without extracapsular extension [7]. For treatment without dissection 66Gy in 33 fractions (BED Gy_10_ 79) is recommended for clinically evident disease and 50Gy in 25 fractions (BD Gy_10_ 60) for subclinical high risk nodes. These data are referenced solely on head and neck site reports [2, 8].

Radiation dose, fractionation, and treatment technique were not prospectively standardised in our retrospective series. The high rate of relapse might be attributed to doses on average lower than recommended by the NCCN guidelines, however a dose-response was not found for local control in this series. The advanced average age and high rate of ECOG performance status > 1 (50%) and grade 3 acute skin toxicity rate approximating 10% argue against an expectation that the NCCN guidelines can be routinely applied to most of these patients.

One prior study by Fogarty et al addresses the issue of radiotherapy dose and technique for post-operative axillary irradiation in high risk skin cancer, which included 24 patients with metastatic cSCC, metastatic melanoma, and merkel cell carcinoma [9]. The deficiency in evidence on the optimal radiotherapy dose was highlighted. The dose recommended by the authors for nodal cutaneous SCC was 50Gy in 25 fractions at five fractions per week. Bolus was applied over scars and surgical drain sites in all patients. The target volume included the axillary operative bed and at risk contiguous supraclavicular nodes. Nodal volumes were defined by contouring the space surrounding the neurovascular bundles of the axillary and supraclavicular vessels in order to obtain a clinical target volume (CTV), with a further 1 cm expansion to obtain a planning target volume (PTV). Of 24 patients audited, 11 of unspecified histology were treated radically with this technique to a dose of > 48Gy with only 1 of 11 failing in-field with a mean follow-up of 20 months. Two additional radical patients not complying with their recommended technique failed in-field. Outcomes for patients treated to lesser doses were not described. It is tempting to ascribe the excellent regional control to compliance with the recommended technique, but the lack of detail regarding histologies and short follow-up make it impossible to draw firm conclusions regarding efficacy for cSCC metastatic to the axilla.

The majority of patients in our series were treated prior to the advent of routine fine cut CT planning in our centre. However, our axillary fields complied with the anatomical borders of the example digital radiograph (if the supraclavicular nodes were intended to be included) in the Fogarty et al series. A notable example of the impact of radiotherapy planning quality on local control for locally advanced SCC of the head and neck is the TROG 02.02 trial, but this actually demonstrated that dose volume compliance was important, not just volumes [10].

Even if our volumes provided adequate coverage, the use of CT planning to optimise dosimetry will reduce long term toxicity as well as ensure dose to the target. This will be particularly relevant in the setting of concurrent chemoradiotherapy or other radiosensitisers.

Though the majority of patients in our study were treated using parallel opposed fields, incorporation of the SCF and the use of bolus varied.

A retrospective study of 200 patients treated with axillary radiotherapy for metastatic melanoma showed that extended field radiotherapy (axilla and SCF) resulted in significantly higher rates of radiotherapy related complications compared with axilla radiation alone [11]. Various regimens have been employed in adjuvant axillary radiotherapy for regional metastatic melanoma, with doses ranging from 50-60Gy in 25 - 30 fractions to 30Gy in five fractions or 24Gy in three fractions in key studies [12, 13]. Acceptable toxicity was reported but a higher frequency of lymphoedema has been observed with increasing fraction size [9, 12, 13]. Our small numbers may have prevented us from demonstrating conclusively correlation of field size with worse acute or late toxicity, particularly lymphoedema. It is clear that failure of local control is generally associated with lymphedema and plexopathy symptoms with only 1 patient developing these symptoms after radiotherapy in the absence of local failure. Moreover, our results do not support routine treatment of both the axilla and SCF in preference to intensification of therapy to the axilla alone, since the larger field size was associated with greater toxicity, and there were no SCF recurrences in the curative group, irrespective of whether the supraclavicular fossa was irradiated.

Although the use of bolus was not shown to influence local recurrence rates in our study, the initial decision to use bolus may have been prompted by the presence of skin involvement or positive margins, both of which may have had a negative impact on local control. In a study of 75 patients treated with radiotherapy following parotidectomy for cSCC metastatic to the parotid, local failure rates were higher in those treated with bolus [14]. This was attributed to worse treatment related toxicity with the use of bolus leading to a greater number of treatment interruptions. A randomized comparison would be required to determine the benefit or otherwise of bolus use.

Despite this being the largest series to date, our sample is still small and did not find significant associations between our selected variables and locoregional control. Well documented predictors of locoregional control and survival include the presence of positive margins or extranodal spread, increasing nodal size, and multiple nodes [1, 15-17]. Most of our patients had poor prognostic features in terms of nodal size and poor differentiation.

In our series, the predominant pattern of treatment failure was locoregional, with a minority of patients experiencing isolated distant failure. Head and neck cSCC studies also confirm locoregional failure as the dominant site of first recurrence, comprising 75-80% of initial failures, with distant metastases as a first site of recurrence occurring in only 20-25% [2, 17, 18]. Distant recurrences however, have been shown to occur more commonly in association with locoregional failure [18]. Almost half the patients with distant failure in our study had simultaneous local failure.

The limited literature for patterns of failure for axillary presentations for cSCC is inconsistent. A recent Australian series including axilla and inguinal cSCC metastases treated by surgery (n = 13) or surgery and radiotherapy (n = 13 median dose 50Gy) reported a crude relapse rate of 38% in irradiated patients with a median follow up of 18.5 months. The rate of local (nodal basin) recurrence was not specified but half of relapses were lung as the first site [19].

Our rate of relapse compares favourably to the series of 24 patients with nodal involvement from limb or truncal cSCC reported by Mullen with a 5 year overall survival of 42% and relapse free survival of 26% but apparently no failures within the dissected nodal basin [20]. Nineteen of these received radiotherapy in addition to surgery but no radiotherapy details were provided. Shaw reported 2 of 8 cSCC failing locally after axillary dissection, with an unknown number of these patients also undergoing radiotherapy [21].

Overall it seems axillary presentation of cSCC has a poor prognosis. As such, strategies aimed at intensification of local therapy may be warranted in order to improve locoregional control rates, including altered fractionation regimens or concurrent use of radiosensitisers. Moreover, the fact that < 10% of these failures was able to be successfully salvaged supports the approach that these patients should be treated aggressively initially.

Post-operative radiotherapy in metastatic cSCC to head and neck nodes has been shown to improve locoregional control rates by 15-20%, and has been established as the standard of care in these patients [2]. Several series have reported inferior outcomes with single modality treatment [22-27]. Veness, in a series of 74 patients with metastatic cutaneous SCC to cervical lymph nodes reported a significant improvement in 5-year disease free survival rates with surgery and adjuvant radiotherapy compared with surgery alone (73% vs 18%, P = 0.001), as well as superior locoregional control (77% vs 15%) [8, 18, 26]. Single modality treatment was also among the independent predictors of worse survival [26]. Although patients treated with axilla surgery alone were not included in our study, the few patients treated with radiotherapy alone had poor cancer control. Locoregional recurrence rates in patients with metastatic cSCC to regional nodes treated with surgery alone have been reported to range from 17-54% [2, 23-26]. Similarly, patients treated with radiotherapy alone can expect locoregional recurrence rates as high as 50% [8, 25, 26]. Audet reported significantly worse disease-specific survival in patients treated with radiotherapy alone compared with surgery and adjuvant radiotherapy [27]. Current research is focused on the benefits of systemic therapy and targeted agents in combination with adjuvant radiotherapy for metastatic cSCC, as has been the experience with mucosal head and neck SCC. The Trans Tasman Radiation Oncology Group is currently running a phase III randomised trial comparing standard adjuvant radiotherapy (60-66Gy in 2Gy per fraction, 5 days per week) with standard radiotherapy and concurrent weekly carboplatin (TROG 05.01) [28]. Cetuximab, a human and mouse chimeric antibody against epidermal growth factor receptor (EGFR), is currently approved for use in treatment of SCCs of the upper aerodigestive tract, which richly express EGFR [29]. Cetuximab added to radiotherapy improves local control and survival in Head and Neck mucosal SCC [30]. The benefits of cetuximab in treatment of recurrent cSCC have been reported in small series of patients [31, 32]. A recent prospective study of 36 patients with chemotherapy-naive unresectable cSCC treated with weekly cetuximab for a minimum of six weeks, reported a response rate of 28% (eight partial and two complete responses) [33]. Since individuals with cSCC are often elderly or immunosuppressed, and difficulties may be encountered in delivering radiotherapy concurrently with chemotherapy, the use of targeted agents such as Cetuximab deserves further exploration.

As well as attempting treatment intensification for presentations with macroscopic axillary involvement, consideration should be given to sentinel node biopsy in higher risk patients. Well reported high risk features of cSCC associated with increased risk of regional nodal metastasis include site (lips, nose, temple, ear, scalp, dorsal hands, anal, genitalia), tumor thickness beyond 5 mm, increasing size (> 20 mm), high grade, perineural and lymphovascular invasion, prior recurrence and immunosuppression [1, 3, 6, 16-18].

A recent landmark paper indicated thickness and desmoplastic growth (lymphovascular or perineural permeation) were the best predictors of elevated nodal failure [34]. Whether dissection or radiotherapy or combination treatment would be required for patients with such high risk features needs to be defined, but it is reasonable to anticipate better outcomes with treatment of subclinical disease [35].

### Conclusion

The available evidence suggests that best outcomes in patients with nodal metastases from cSCC are achieved with surgery and adjuvant radiotherapy, however these remain suboptimal. The prevalence of locoregional failure and the low success rates of salvage therapy suggest that intensification of local therapy could lead to significant improvements in locoregional control and survival, as could better identification of subclinical nodal involvement in high risk patients.
